# The effect of post-injection ^18^F-FDG PET scanning time on texture analysis of peripheral nerve sheath tumours in neurofibromatosis-1

**DOI:** 10.1186/s13550-017-0282-3

**Published:** 2017-04-20

**Authors:** Eitan Lovat, Musib Siddique, Vicky Goh, Rosalie E. Ferner, Gary J. R. Cook, Victoria S. Warbey

**Affiliations:** 1 Guy’s, King’s and St Thomas’ School of Medicine, King’s College London, London, UK; 2 Department of Cancer Imaging, Division of Imaging Sciences and Biomedical Engineering, King’s College London, London, UK; 3 National Neurofibromatosis Service, Department of Neurology, Guys and St Thomas’ NHS Foundation Trust, London, UK; 4 Clinical PET Centre, Division of Imaging Sciences and Biomedical Engineering, King’s College London, St Thomas’ Hospital, London, SE1 7EH UK

**Keywords:** Texture features, Neurofibromatosis-1, ^18^F-FDG PET/CT

## Abstract

**Background:**

Texture features are being increasingly evaluated in ^18^F-fluorodeoxyglucose positron emission tomography (^18^F-FDG PET) as adjunctive imaging biomarkers in a number of different cancers. Whilst studies have reported repeatability between scans, there have been no studies that have specifically investigated the effect that the time of acquisition post-injection of ^18^F-FDG has on texture features. The aim of this study was to investigate if texture features change between scans performed at different time points post-injection.

**Results:**

Fifty-four patients (30 male, 24 female, mean age 35.1 years) with neurofibromatosis-1 and suspected malignant transformation of a neurofibroma underwent ^18^F-FDG PET/computed tomography (CT) scans at 101.5 ± 15.0 and 251.7 ± 18.4 min post-injection of 350 MBq ^18^F-FDG to a standard clinical protocol. Following tumour segmentation on both early and late scans, first- (*n* = 37), second- (*n* = 25) and high-order (*n* = 31) statistical features, as well as fractal texture features (*n* = 6), were calculated and a comparison was made between the early and late scans for each feature.

Of the 54 tumours, 30 were benign and 24 malignant on histological analysis or on clinical follow-up for at least 5 years. Overall, 25/37 first-order, 9/25 second-order, 13/31 high-order and 3/6 fractal features changed significantly (*p* < 0.05) between early and late scans. The corresponding proportions for the 30 benign tumours alone were 22/37, 7/25, 8/31 and 2/6 and for the 24 malignant tumours, 11/37, 6/25, 8/31 and 0/6, respectively.

**Conclusions:**

Several texture features change with time post-injection of ^18^F-FDG. Thus, when comparing texture features in intra- and inter-patient studies, it is essential that scans are obtained at a consistent time post-injection of ^18^F-FDG.

## Background

There is an assumption that medical images contain additional data that is not apparent to the human eye and the field of radiomics aims to extract this information through (semi) automated analysis, without the need to change the image acquisition protocols [1, 2]. Texture analysis, measuring image heterogeneity, is an example of additional information that is contained within medical images. Although first-order statistics, based on global measures from voxel intensity histograms, are not a true measure of image texture, they are often reported due to their clinical relevance in a number of studies. Second-order statistics consider the relationship between pairs of voxels providing a measure of local texture features. High-order statistics consider the relationship between three or more voxels and provide a measure of both local and regional texture features. Fractal analysis is a further method that can be used to quantify texture information on the basis of repeating geometric patterns (self-similarity) and roughness [3–5].

Whilst a number of factors have been described that influence the measurement, accuracy and reproducibility of texture features [4, 6, 7], to our knowledge, there have been no published data on how texture features change with time post-injection of ^18^F-FDG in soft tissue tumours. As there is a growing interest in using texture features in the clinical environment, these data are essential to inform on the design of clinical and research protocols to enable intra/inter-patient scans to be compared reliably and multi-centre trials to be carried out.

Standardised uptake value (SUV) parameters have been shown to change with time post-injection of ^18^F-FDG [8–11], and we hypothesised that texture parameters may also change with time. Therefore, the aim of this study was to investigate the effect of time post-injection of ^18^F-FDG on the measurement of texture features in a cohort of patients with neurofibromatosis-1 (NF1) in whom malignant transformation of neurofibromas to malignant peripheral nerve sheath tumours (MPNST) was suspected clinically and in whom ^18^F-FDG PET data had been acquired at two separate time points post-injection. As a purely technical study, we did not aim to assess the ability of texture features to discriminate benign from MPNSTs, an analysis that will be the subject of a separate study.

## Methods

### Patients

This retrospective study included 54 patients with NF1 attending our national neurofibromatosis service (30 male, 24 female, mean age 35.1 years). All patients had symptomatic plexiform neurofibromas clinically suspected of malignant transformation and were referred for further investigation with ^18^F-FDG PET/CT. An institutional review board waiver was obtained for this retrospective analysis. All patients either had histological confirmation of the tumours or were followed clinically for at least 5 years.

### ^18^F-FDG PET/CT scan acquisition and analysis

Adult patients were injected with 350 (±10%) MBq of ^18^F-FDG, and in children, the injected activity was scaled by body weight (weight/70 × 350 MBq). All patients had blood-glucose levels below 10 mmol/l at the time of injection. Imaging was performed at two time points post-injection of ^18^F-FDG: an initial early acquisition at 101.5 ± 15.0 min and a later acquisition at 251.7 ± 18.4 min, as per the standard clinical protocol of our department for characterisation of masses in patients with NF1 [8]. The early scan was from the cerebellum to mid-thigh with additional images acquired if the tumour was below the mid-thigh or above the cerebellum. The late scan involved acquiring a local view of the symptomatic tumour only. The acquisition time for both the early and late scans was 5 min per bed position.

Scans were performed on one of two scanners (Discovery VCT or DST, GE Healthcare, Chicago, USA) which are cross-calibrated to within 3% [12]. All images, from both scanners, were reconstructed with a voxel size of 4.7 mm and slice thickness of 3.27 mm using the ordered-subset expectation maximisation algorithm (2 iterations, 20 subsets). They were subsequently post-filtered using a 3D Gaussian kernel with a full-width at half maximum of 6 mm. Low-dose CT was acquired at 120 kVp and 65 mAs for the purposes of anatomical localisation and attenuation correction without administration of oral or intravenous contrast agent.

**Fig. 1:**
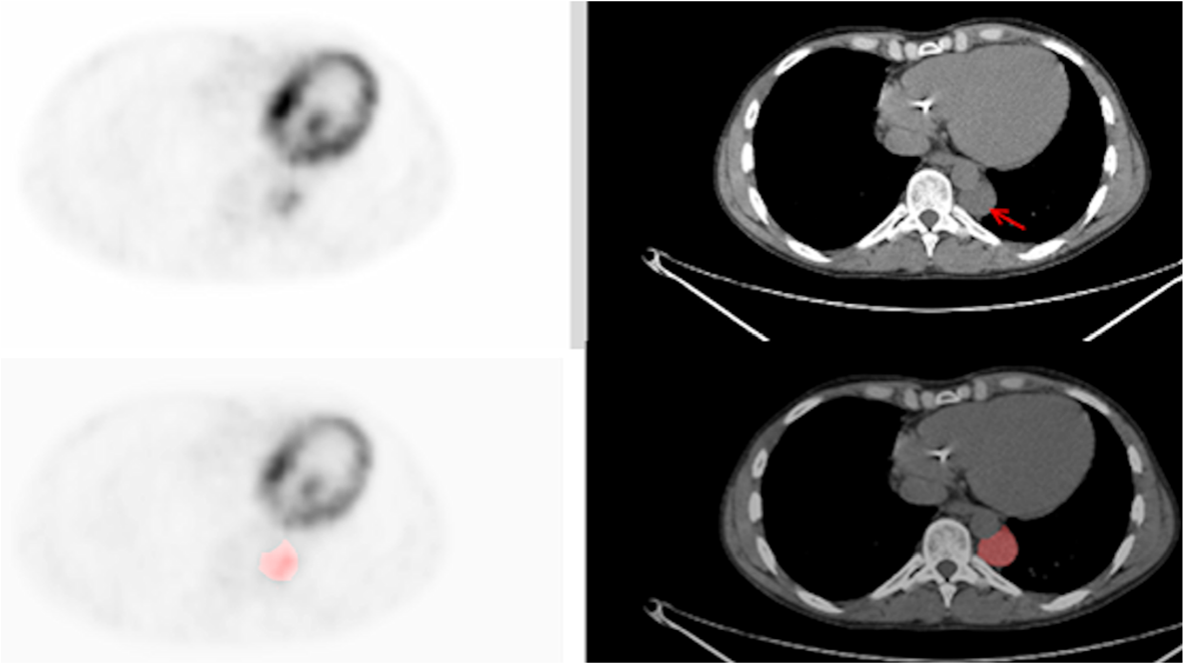
Axial ^18^F-FDG PET and CT scans. The tumour is indicated by the *arrow* (*above*). A region of interest is shown on the same PET and CT slice of the tumour (*below*)

The reconstructed PET datasets were imported into in-house texture analysis software implemented in MATLAB (Release 2013b, The MathWorks, Inc., Natick, MA, USA). Many of the tumours, particularly those that were classified as benign, showed only very low-grade ^18^F-FDG uptake, and it was therefore not possible to implement automated segmentation (e.g., threshold defined by a percentage of maximum standardised uptake value (SUVmax) or a fuzzy locally adapted Bayesian (FLAB) method) nor was it possible to reliably define the region of interest (ROI) by hand on the PET scan. All tumours, at both time points, were therefore segmented manually on the CT images where the tumour edges were easily defined. ROI definition was carried out by an experienced operator trained in both radiology and nuclear medicine. The ROIs drawn on the CT scans were automatically mapped onto the PET scan (Fig. 1). Statistical and textural features that were calculated from tumour volumes of interest included 37 first-order, 25 second-order, 31 high-order and 6 fractal features as listed in Table 1. First-order ROI features were decay corrected from the time of injection. Voxel values within the tumour volume of interest (VOI) were resampled to yield 64 discrete equally spaced bins. Seven 3D direction vectors and 2 distances were considered resulting in 14 matrices. The 2 distances were used to capture relationships between voxels at larger distances and 7 directions to optimise computational time. The texture descriptors were obtained from each matrix followed by averaging the values calculated separately in each matrix. Fractal features were computed using a differential box-counting method.

**Table 1:** Early and late scan median values (and range) of all parameters

Texture feature	Early scan median value—all tumours (range)	Late scan median value—all tumours (range)	↑ or ↓ (all tumours)	*p* value early vs late (all tumours)	Correlation early vs late—all tumours (*r* and (*p*) values)	↑ or ↓ (benign)	*p* value early vs late (benign)	↑ or ↓ (malignant)	*p* value early vs late (malignant)
First-order texture features									
ROI: mean	6226.9 (918.2–4039.2)	5773.3 (2646.8–55066.4)	–	0.19	0.81 (<0.001)	↓	0.011	–	0.8
ROI: maximum	12881.2 (3571.6–120766.4)	15689.9 (4654.7–221719.7)	↑	0.02	0.86 (<0.001)	–	0.15	–	0.081
ROI: minimum	1906.4 (157.1–15271.0)	1119.8 (154.7–15198.9)	↓	0.001	0.53 (<0.001)	↓	0.001	–	0.19
ROI: range	11757.5 (2135.7–119645.4)	14371.1 (2848.3–220643.8)	↑	0.002	0.87 (<0.001)	↑	0.01	↑	0.043
ROI: standard deviation	2177.4 (444.2–19219.2)	2199.0 (502.3–26481.3)	↑	0.015	0.86 (<0.001)	–	0.11	–	0.076
ROI: skewness	0.95 (−0.93–4.54)	3.04 (0.25–13.3)	↑	<0.001	0.05 (0.7)	↑	<0.001	↑	<0.001
ROI: kurtosis	5.5 (3.7–26.2)	15.6 (10.1–71.1)	↑	<0.001	0.4 (0.003)	↑	<0.001	↑	<0.001
ROI: coefficient of variation	0.57 (0.26–1.14)	1.72 (0.73–3.31)	↑	<0.001	0.65 (<0.001)	↑	<0.001	↑	<0.001
ROI: median	5895.6 (833.1–37490.6)	5324.8 (2425.3–50631.2)	–	0.07	0.79 (<0.001)	↓	0.002	–	0.98
ROI: 10th percentile	3822.0 (506.6–18913.7)	2692.1 (1295.6–24466.4)	↓	0.002	0.63 (<0.001)	↓	<0.001	–	0.35
ROI: 25th percentile	4787.5 (637.2–24843.1)	3966.1 (1817.0–33982.6)	↓	0.008	0.72 (<0.001)	↓	<0.001	–	0.69
ROI: 75th percentile	7148.3 (1074.9–52427.0)	7174.7 (3026.9–71854.5)	–	0.44	0.85 (<0.001)	↓	0.041	–	0.69
ROI: 90th percentile	8691.3 (1414.5–68876.1)	8656.2 (3444.4–94054.6)	–	0.97	0.87 (<0.001)	–	0.17	–	0.38
ROI: entropy	5.75 (4.51–6.01)	5.71 (4.55–6.01)	–	0.34	0.53 (<0.001)	–	0.98	–	0.12
ROI: energy	0.0038 (0.0028–0.0397)	0.004 (0.0027–0.013)	–	0.53	0.52 (<0.001)	–	0.83	–	0.20
ROI: LoG entropy 1_5	5.12 (4.12–5.44)	5.02 (3.87–5.43)	↓	0.002	0.6 (<0.001)	↓	0.016	↓	0.033
ROI: LoG entropy 2_0	5.14 (4.22–5.44)	5 (3.87–5.41)	↓	0.004	0.57 (<0.001)	–	0.052	↓	0.04
ROI: LoG entropy 2_5	5.15 (4.39–5.49)	5.11 (4.05–5.49)	–	0.071	0.61 (<0.001)	–	0.39	–	0.063
Metabolic active volume (cm^3^)	35.6 (3.3–303.9)	34.9 (3.5–301.4)	–	0.069	0.89 (<0.001)	–	0.39	–	0.29
Effective diameter (cm)	4.08 (1.84–8.34)	4.05 (1.98–8.32)	–	0.14	0.89 (<0.001)	–	0.59	–	0.29
SUV: mean	1.42 (0.5–8.89)	1.27 (0.41–9.1)	↓	0.006	0.94 (<0.001)	↓	<0.001	–	0.89
SUV: total lesion glycolysis (TLG)	62.1 (2.6–2412.7)	47.2 (2.3–2321.5)	↓	0.005	0.92 (<0.001)	↓	<0.001	–	0.55
SUV: peak	2.5 (0.7–23.4)	2.3 (0.7–23.3)	–	0.93	0.92 (<0.001)	↓	0.032	–	0.30
SUV: maximum	3.1 (0.9–30.8)	3.5 (0.8–30.8)	↑	0.036	0.93 (<0.001)	–	0.72	↑	0.039
SUV: minimum	0.45 (0.09–3.35)	0.26 (0.03–3.27)	↓	<0.001	0.56 (<0.001)	↓	<0.001	–	0.15
SUV: range	2.6 (0.6–30.5)	3.2 (0.7–30.5)	↑	0.002	0.92 (<0.001)	–	0.079	↑	0.019
SUV: standard deviation	0.45 (0.12–5.05)	0.44 (0.12–5.12)	↓	0.033	0.93 (<0.001)	–	0.704	–	0.092
SUV: skewness	0.54 (−0.52–2.54)	0.67 (−0.01–2.92)	↑	0.002	0.05 (0.7)	↑	0.037	↑	0.014
SUV: kurtosis	3.1 (2.1–14.9)	3.4 (2.2–15.6)	↑	0.003	0.4 (0.003)	–	0.090	↑	0.005
SUV: coefficient of variation	0.31 (0.15–0.65)	0.38 (0.16–0.73)	↑	<0.001	0.65 (<0.001)	↑	0.001	↑	0.034
SUV: median	1.4 (0.5–8.4)	1.1 (0.4–8.7)	↓	0.001	0.92 (<0.001)	↓	<0.001	–	0.61
SUV: 10th percentile	0.9 (0.3–3.9)	0.7 (0.3–3.9)	↓	<0.001	0.74 (<0.001)	↓	<0.001	–	0.12
SUV: 25th percentile	1.1 (0.4–4.9)	0.9 (0.3–5.4)	↓	<0.001	0.84 (<0.001)	↓	<0.001	–	0.21
SUV: 75th percentile	1.8 (0.6–12.3)	1.5 (0.5–12.6)	↓	0.043	0.95 (<0.001)	↓	<0.001	–	0.73
SUV: 90th percentile	2.1 (0.7–15.8)	1.9 (0.6–16.1)	–	0.45	0.95 (<0.001)	↓	0.001	–	0.35
SUV: entropy	0.8 (0.17–2.98)	0.81 (0.12–2.99)	↑	0.033	0.85 (<0.001)	–	0.3	–	0.092
SUV: energy	0.5 (0.06–0.92)	0.5 (0.05–0.95)	–	0.053	0.83 (<0.001)	–	0.13	–	0.17
Second-order texture features									
GLCM: autocorrelation	202.1 (50.8–432.5)	152.6 (29.6–295.2)	↓	<0.001	0.26 (0.6)	↓	0.003	↓	0.026
GLCM: cluster prominence	39992.1 (74471.1–118471.8)	33630.5 (4478.5–113080.7)	–	0.23	0.64 (<0.001)	–	0.2	–	0.73
GLCM: cluster shade	541.9 (−305.0–2242.1)	630.5 (−85.7–2134.6)	–	0.13	0.44 (0.001)	–	0.32	–	0.19
GLCM: contrast	36.3 (8.4–140.1)	41.2 (5.4–146.2)	–	0.39	0.58 (<0.001)	–	0.17	–	0.86
GLCM: correlation	0.99 (0.96–0.99)	0.98 (0.95–0.99)	↓	0.01	0.68 (<0.001)	–	0.31	↓	0.001
GLCM: difference entropy	2.56 (1.87–3.14)	2.62 (1.66–3.12)	–	0.83	0.57 (<0.001)	–	0.47	–	0.71
GLCM: difference variance	36.3 (8.4–140.1)	41.2 (5.4–146.2)	–	0.39	0.58 (<0.001)	–	0.17	–	0.86
GLCM: dissimilarity	4.71 (2.05–10.04)	5.04 (1.59–11.24)	–	0.93	0.6 (<0.001)	–	0.32	–	0.42
GLCM: energy	0.020 (0.008–0.06)	0.023 (0.007–0.1)	↑	0.016	0.5 (<0.001)	↑	0.033	–	0.25
GLCM: entropy	5.83 (4.39–6.34)	5.79 (4.17–6.44)	↓	0.016	0.47 (<0.001)	–	0.090	–	0.097
GLCM: homogeneity	0.73 (0.53–0.89)	0.73 (0.5–0.91)	–	0.30	0.67 (<0.001)	–	0.12	–	0.80
GLCM: information measure correlation 1	−0.06 (−0.15--0.01)	−0.05 (−0.17--0.01)	–	0.069	0.69 (<0.001)	↑	0.047	–	0.48
GLCM: information measure correlation 2	0.99 (0.96–0.99)	0.98 (0.95–0.99)	↓	0.005	0.62 (<0.001)	–	0.23	↓	0.002
GLCM: inverse difference moment	0.73 (0.53–0.89)	0.73 (0.5–0.91)	–	0.30	0.67 (<0.001)	–	0.12	–	0.80
GLCM: inverse difference moment normalised	0.9992 (0.9974–0.9998)	0.9992 (0.9965–0.9998)	–	0.91	0.7 (<0.001)	–	0.31	–	0.28
GLCM: inverse difference normalised	0.98 (0.96–0.99)	0.98 (0.96–0.99)	–	0.48	0.68 (<0.001)	–	0.17	–	0.59
GLCM: maximum probability	0.05 (0.02–0.13)	0.05 (0.01–0.2)	–	0.081	0.42 (0.001)	–	0.15	–	0.36
GLCM: sum average	27.7 (13.1–40.9)	23.1 (9.8–33.5)	↓	<0.001	0.22 (0.1)	↓	0.001	↓	0.026
GLCM: sum entropy	3.71 (3.02–3.96)	3.65 (2.88–4.0)	↓	0.004	0.51 (<0.001)	↓	0.035	–	0.052
GLCM: sum of squares variance	221.2 (53.1–438.1)	160.2 (30.6–313.2)	↓	<0.001	0.27 (0.046)	↓	0.002	↓	0.034
GLCM: sum variance	654.4 (133.8–1468.2)	466.3 (72.8–984.9)	↓	0.001	0.25 (0.064)	↓	0.002	↓	0.028
GLDM: mean	4.71 (2.05–10.04)	5.04 (1.59–11.24)	–	0.93	0.6 (<0.001)	–	0.32	–	0.42
GLDM: entropy	2.56 (1.87–3.14)	2.62 (1.66–3.12)	–	0.83	0.57 (<0.001)	–	0.47	–	0.71
GLDM: variance	13.5 (4.2–39.3)	15.8 (2.9–64.6)	–	0.21	0.59 (<0.001)	–	0.21	–	0.65
GLDM: contrast	36.3 (8.4–140.1)	41.2 (5.4–146.2)	–	0.39	0.58 (<0.001)	–	0.17	–	0.86
High-order texture features									
GLRL: short run emphasis	0.29 (0.17–0.71)	0.29 (0.16–0.77)	↑	0.007	0.8 (<0.001)	–	0.090	↑	0.034
GLRL: long run emphasis	209.6 (66.8–415.0)	164.6 (41.9–280.1)	↓	<0.001	0.22 (0.11)	↓	0.003	↓	0.015
GLRL: grey-level nonuniformity	8082.9 (975.1–73005.3)	7639.5 (991.0–65663.1)	–	0.54	0.88 (<0.001)	–	0.83	–	0.67
GLRL: run length nonuniformity	917.6 (158.9–6079.5)	947.5 (147.8–6787.9)	↑	0.023	0.87 (<0.001)	–	0.10	–	0.12
GLRL: run percentage	0.91 (0.65–0.98)	0.91 (0.59–0.99)	–	0.17	0.75 (<0.001)	–	0.24	–	0.53
GLRL: low grey-level run emphasis	0.93 (0.76–0.98)	0.93 (0.7–0.99)	–	0.31	0.75 (<0.001)	–	0.29	–	0.71
GLRL: high grey-level run emphasis	40.9 (7.4–425.5)	35.4 (7.42–315.5)	–	0.87	0.86 (<0.001)	–	0.70	–	0.93
GLRL: short run low grey-level emphasis	0.08 (0.04–0.18)	0.09 (0.04–0.17)	↑	0.027	0.61 (<0.001)	–	0.34	↑	0.024
GLRL: short run high grey-level emphasis	40.0 (4.4–424.2)	34.2 (5.5–314.2)	–	0.94	0.87 (<0.001)	–	0.80	–	0.95
GLRL: long run low grey-level emphasis	184.9 (44.6–377.7)	141.0 (26.0–251.9)	↓	<0.001	0.18 (0.19)	↓	0.004	↓	0.021
GLRL: long run high grey-level emphasis	924.6 (279.4–3387.9)	711.4 (336.0–2341.7)	↓	<0.001	0.66 (<0.001)	↓	0.003	↓	0.002
GLRL: intensity variability	945001.0 (20680.9–68924026.0)	921355.2 (22561.0–56722816.4)	–	0.46	0.87 (<0.001)	–	0.3	–	0.78
GLRL: run length variability	92938.6 (3597.7–5739641.7)	103541.0 (3494.2–4675872.2)	↑	0.016	0.86 (<0.001)	↑	0.008	–	0.28
GLSZM: short zone emphasis	0.02 (0.01–0.08)	0.02 (0.01–0.06)	–	0.47	0.44 (0.001)	–	0.41	–	0.93
GLSZM: long zone emphasis	253.9 (126.6–384.9)	229.9 (129.6–327.8)	↓	0.015	0.36 (0.007)	↓	0.035	–	0.18
GLSZM: intensity nonuniformity	97.4 (22.7–654.4)	128.9 (33.11–594.0)	–	0.18	0.61 (<0.001)	–	0.2	–	0.46
GLSZM: zone length nonuniformity	25.6 (10.2–180.1)	35.8 (10.2–200.0)	–	0.28	0.72 (<0.001)	–	0.34	–	0.65
GLSZM: zone percentage	0.068 (0.018–0.313)	0.071 (0.018–0.335)	↑	0.006	0.79 (<0.001)	–	0.075	↑	0.028
GLSZM: low-intensity zone emphasis	0.39 (0.28–0.54)	0.41 (0.31–0.6)	↑	0.001	0.63 (<0.001)	↑	0.001	–	0.29
GLSZM: high-intensity zone emphasis	4108.4 (45.4–43861.1)	4043.2 (31.4–38723.0)	↓	0.049	0.79 (<0.001)	–	0.63	↓	0.037
GLSZM: short zone low-intensity emphasis	0.0048 (0.0016–0.0535)	0.0053 (0.0017–0.0355)	–	0.83	0.52 (<0.001)	–	0.25	–	0.46
GLSZM: short zone high-intensity emphasis	33.0 (1.5–3423.9)	72.9 (0.45–4921.8)	–	0.59	0.74 (<0.001)	–	0.59	–	0.73
GLSZM: long zone low-intensity emphasis	100.8 (56.8–150.4)	104.3 (54.2–167.0)	–	0.40	0.34 (0.11)	–	0.59	–	0.51
GLSZM: long zone high-intensity emphasis	599829.4 (2743.9–11949368.6)	322428.3 (5079.9–3059584.4)	↓	<0.001	0.81 (<0.001)	↓	0.001	↓	0.016
GLSZM: intensity variability	231.6 (29.5–3815.9)	353.3 (50.2–3591.3)	–	0.19	0.62 (<0.001)	–	0.18	–	0.57
GLSZM: size zone variability	56.2 (13.8–869.2)	101.9 (17.3–1066.3)	–	0.26	0.67 (<0.001)	–	0.19	–	0.69
NGTDM: coarseness	7.1 (4.3–17.4)	6.6 (4.0–15.3)	–	0.087	0.79 (<0.001)	–	0.48	–	0.092
NGTDM: contrast	0.14 (0.02–0.59)	0.12 (0.01–0.46)	–	0.16	0.53 (<0.001)	–	0.31	–	0.33
NGTDM: busyness	4.2 (0.7–32.9)	4.7 (0.8–33.3)	↑	0.007	0.89 (<0.001)	↑	0.006	–	0.2
NGTDM: complexity	0.09 (0.01–2)	0.11 (0–1.55)	–	0.66	0.89 (<0.001)	–	0.94	–	0.59
NGTDM: texture strength	0.33 (0.03–1.93)	0.35 (0.04–1.84)	–	0.086	0.83 (<0.001)	–	0.066	–	0.48
Model-based texture features									
FD: fractal dimension mean	3.23 (2.85–4.14)	3.23 (2.85–4.09)	–	0.67	0.87 (<0.001)	–	0.89	–	0.65
FD: SD	0.37 (0.2–1.31)	0.44 (0.22–1.92)	↑	0.018	0.74 (<0.001)	–	0.063	–	0.15
FD: lacunarity	0.02 (0–0.73)	0.02 (0.01–0.63)	↑	0.006	0.72 (<0.001)	↑	0.003	–	0.44
FD: Hurst exponent	0.33 (0.06–1.62)	0.34 (0.03–1.69)	–	0.73	0.55 (<0.001)	–	0.21	–	0.39
FD: blanket mean	3.91 (2.66–4.98)	3.64 (2.65–4.89)	↓	0.014	0.69 (<0.001)	↓	0.009	–	0.49
FD: inverse	0.31 (0.24–0.35)	0.31 (0.24–0.35)	–	0.85	0.77 (<0.001)	–	0.83	–	0.99

For significant differences between early and late scans, an increase (↑) or decrease (↓) in parameter value with time post-injection is shown. ROI: LoG entropy 1_5, 2_0 and 2_5 represent entropy values calculated for a ROI after applying Laplacian of Gaussian (LoG) transformation with a point spread function of standard deviation 1_5, 2_0, 2_5 mm

*ROI* region of interest, *LoG* Laplacian of Gaussian, *SUV* standardised uptake value, *FD* fractal dimension, *GLCM* grey-level co-occurrence matrix, *GLDM* grey-level difference matrix, *GLRL* grey-level run length matrix, *GLSZM* grey-level size zone matrix, *NGTDM* neighbourhood grey-tone difference matrix

To assess inter-observer variability, a random subset of 16 patients had VOIs defined on early and late scans by a separate operator blinded to the initial observer measurements and clinical data.

### Statistical methods

All statistical analyses were performed using IBM ® SPSS predictive analytics software, v22.0.0.0. As data were not normally distributed, non-parametric tests were performed. For each texture feature, the values obtained were compared between the initial and later time points using the related-samples Wilcoxon signed-rank test and correlations made with Spearman correlation. Comparisons were performed for all 54 tumours and for the subsets of the 30 benign and the 24 malignant tumours. A significance level of *p* < 0.05 was used. Inter-observer variation was assessed with intraclass correlation coefficients (ICC).

## Results

Good inter-observer agreement was found for measurement of all parameters with mean ICC scores for individual patient scans of 0.93 and 0.96 for early and late scanning time points, respectively, and there was no significant difference between the segmented tumour volumes obtained from the early and late scans (median 35.6 vs 34.9 cm^3^, respectively; *p* = 0.069). High correlation was observed between early and late scan data for most texture features (mean *r* value = 0.66 ± 0.21; Table 1).

Several first-, second-, high-order statistical and fractal features were significantly different between early and late scans (*p* < 0.05). In summary, overall 25/37 (68%) first-order, 9/25 (36%) second-order, 13/31 (42%) high-order and 3/6 (50%) fractal features showed significant changes, i.e. 50/99 (50%) parameters in total (Table 1). For the 30 benign tumours, 22/37 first-order, 7/25 second-order, 8/31 high-order and 2/6 fractal features changed significantly (*p* < 0.05) between the early and late scans. The corresponding numbers for the 24 malignant tumours were 11/37, 6/25, 8/31 and 0/6 (*p* < 0.05) (Table 2). Fifteen of the texture features that changed significantly did so for both benign and malignant tumours whilst the majority of features changed only for benign (*n* = 24) or malignant (*n* = 10) tumours.

**Table 2:** Number of texture features that significantly changed (*p* < 0.05), either increasing (↑) or decreasing (↓) between early and late scanning time points

	First order	Second order	High order	Model based	Total
All 54 tumours	25/37 (12↑, 13↓)	9/25 (1↑, 8↓)	13/31 (7↑, 6↓)	3/6 (2↑, 1↓)	50/99 (22↑, 28↓)
30 benign tumours	22/37 (6↑, 16↓)	7/25 (2↑, 5↓)	8/31 (3↑, 5↓)	2/6 (1↑, 1↓)	39/99 (12↑, 27↓)
24 malignant tumours	11/37 (9↑, 2↓)	6/25 (0↑, 6↓)	8/31 (3↑, 5↓)	0/6 (0↑, 0↓)	25/99 (12↑, 13↓)

Overall, more texture features decreased (27/39 benign; 13/25 malignant) than increased (12/39 benign; 12/25 malignant) with time. This pattern was true for first-order, second-order and high-order features in benign tumours and for second-order and high-order features in malignant tumours. However, in malignant tumours, more first-order features increased (*n* = 9) than decreased (*n* = 2) (Table 2).

## Discussion

Previous researchers have demonstrated changes in measured SUV parameters post-injection of ^18^F-FDG and that this may even be of benefit for differentiating benign and malignant lesions [8–11]. However, to our knowledge, this is the first study that has investigated how global first-order and loco-regional texture features change with time post-injection of ^18^F-FDG.

Our study has demonstrated that a significant number of statistical first-, second- and high-order and model-based fractal features change with time post-injection of ^18^F-FDG in benign and malignant PNSTs. These findings suggest that both global and loco-regional uptake of ^18^F-FDG has not stabilised in both benign and malignant tumours by 101.5 ± 15.0 min after injection. We observed an expected high correlation between early and late scan texture features and the differences would therefore be unlikely to impact significantly on discriminatory ability between benign and malignant tumours but would be of greater importance in studies where serial texture features were being calculated as response measures.

The finding that a greater proportion of the global first-order features changed than second-order, high-order or model-based texture features suggests that global changes predominate over regional or local changes in ^18^F-FDG distribution. However, a significant proportion of second-order, high-order and fractal texture features also changed, showing that regional and local redistribution of ^18^F-FDG also occurs with time.

For first-order features, as expected, SUVmax increased with time for malignant but not benign tumours, as previously described for a number of malignant tumours [8–11].

Overall, SUVmean decreased with time, predominantly due to a decrease in uptake in benign tumours. First-order entropy and standard deviation, reflecting the global tumour randomness and distribution of voxel intensities, increased with time across the whole group of tumours but not in either of the benign or malignant groups alone.

For second-order features that reflect the relationships between pairs of voxel intensities and their spatial distribution, 8 out of 9 of the 25 texture features that changed showed a reduction. GLCM energy, a measure of uniformity, increased, and therefore overall the changes in these local texture features implied a reduction in heterogeneity with time. Second-order features showed a decrease in heterogeneity in both benign and malignant tumours suggesting there is a change in relative ^18^F-FDG distribution in the tumours between the two different time points, causing a change in local tumour texture features.

With high-order textures features, there were increases and decreases in a number of local and regional features in both benign and malignant tumours but with no consistent pattern. This suggests that considering texture features as showing heterogeneity or homogeneity in a binary manner is probably an oversimplification of what each feature represents mathematically. Similarly, a consistent pattern of change was not seen with model-based fractal features.

A number of technical factors are known to affect the measurement and reproducibility of texture features including matrix size, reconstruction parameters, bin width and tumour volume [4, 6, 7]. Our findings demonstrate additionally the importance of quoting post-injection ^18^F-FDG scanning times when discussing texture features and the importance of consistent post-injection ^18^F-FDG scanning times when comparing global and texture features of patient tumours in inter- and intra-patient longitudinal studies.

There are some limitations to our study. We only considered PNSTs in NF1 patients and as such, it is not possible to generalise these findings to other tumour types, and future research should investigate how texture features change over time in other cancers. Scans were acquired at 101.5 ± 15.0 and 251.7 ± 18.4 min post-injection in this study as per the clinical protocol in our department [8]. Therefore, we cannot comment on the detail of the kinetics of change between these time points or on the magnitude of change compared to scans acquired at 60 min post-injection, which is a more commonly used clinical protocol elsewhere. Whilst two scanners were used for data used in this study, the acquisition and reconstruction parameters were identical and knowing that quantitative differences were minimal [12], it is unlikely that this will have introduced a significant bias in results. All the tumours included in this project were segmented manually on the CT component of the PET/CT scan, and therefore ROIs are subject to more variability than semi-automated methods such as threshold-based or FLAB. This was unavoidable as low-grade activity in many of the tumours meant that automated methods and direct ROI placement on the PET images proved impossible. However, even on non-contrast-enhanced CT scans as used in our study, the edges of benign and malignant neurofibromas are usually well demarcated (Fig. 1), thus facilitating ROI definition and VOI definition proved straightforward with good inter-observer reproducibility. This method also has the advantage of minimising differences in segmentation volumes due to changes in ^18^F-FDG distribution, ensuring the whole tumour is included at both time points. The ROIs that were drawn on the CT scan were mapped onto the PET scan. Although all scans were checked qualitatively by an experienced operator to ensure there was no mis-registration of the ROIs, we otherwise made the assumption of accurate co-registration with no patient movement between CT and PET acquisitions.

As the later scans had fewer counts following radioactive decay of ^18^F-FDG, we cannot exclude image noise as an element that may have contributed to differences in texture features. However, more texture features reduced with time (i.e., became more homogeneous) and so it is unlikely that this is a dominant effect. Lastly, the literature suggests that many texture features may be redundant due to collinearity between features and that only a small number of features should be used based on robustness to technical factors and reproducibility [4, 6]. However, as an initial study of the phenomenon of change in texture feature quantification with time, we preferred to report on multiple features with and without known collinearity to document these findings as broadly as possible.

## Conclusions

This study has demonstrated that many ^18^F-FDG PET texture features differ significantly between early and late scan acquisition time points. As such, it is important to scan patients at consistent times when measuring texture features in longitudinal patient studies, especially in multi-centre patient trials.

## Abbreviations

^18^F-FDG PET/CT: ^18^F-fluorodeoxyglucose positron emission tomography/computed tomography
FLAB: Fuzzy locally adapted Bayesian
ICC: Intraclass correlation
MPNST: Malignant peripheral nerve sheath tumour
NF1: Neurofibromatosis-1
ROI: Region of interest
SUVmax: Maximum standardised uptake value
VOI: Volume of interest
